# International evaluation of the psychometrics of health-related quality of life questionnaires for use among long-term survivors of testicular and prostate cancer

**DOI:** 10.1186/s12955-017-0670-4

**Published:** 2017-05-11

**Authors:** Marieke van Leeuwen, Jacobien M. Kieffer, Fabio Efficace, Sophie D. Fosså, Michel Bolla, Laurence Collette, Marc Colombel, Ugo De Giorgi, Bernhard Holzner, Lonneke V. van de Poll-Franse, Hendrik van Poppel, Jeff White, Ronald de Wit, Susanne Osanto, Neil K. Aaronson, for the European Organisation for Research and Treatment of Cancer Quality of Life Group, Genito-Urinary Cancers Group and Radiation Oncology Group, and the NCRN Testis Clinical Studies Group

**Affiliations:** 1 Division of Psychosocial Research and Epidemiology, The Netherlands Cancer Institute-Antoni van Leeuwenhoek Hospital, Plesmanlaan 121, Amsterdam, The Netherlands; 2 Unit, Italian Group for Adult Hematologic Diseases (GIMEMA) Data Center, Via Benevento 6, Rome, Italy; 3 Oslo University Hospital, Radiumhospital, National Advisory Unit on Late Effects after Cancer Treatment, Oslo, Norway; 4 Department of Radiation Oncology, Centre Hospitalier Universitaire A Michallon, BP217, 38043 Grenoble cedex 09, France; 5 Statistics Department, EORTC Headquarters, Brussels, Belgium; 6 Department of General Urology and Oncology, Hôpital Édouard Herriot, 5 Place d’Arsonval, Lyon, France; 7 Department of Medical Oncology, Istituto Scientifico Romagnolo per lo Studio e la Cura dei Tumori (IRST) - IRCCS, Meldola, Italy; 8 Department of Psychiatry, Innsbruck University Hospital, Sternwartestraße 15, Innsbruck, Austria; 9 Comprehensive Cancer Organisation Netherlands (CCCS), Netherlands Cancer Registry, Zernikestraat 29, Eindhoven, The Netherlands; 10 Department of Medical Psychology, Tilburg University, Tilburg, The Netherlands; 11 Department of Urology, University Hospital, K.U. Leuven, Herestraat 49, Leuven, Belgium; 12 The Beatson West of Scotland Cancer Center, Glasgow, Scotland; 13 Department of Medical Oncology, Rotterdam Cancer Institute (Dr Daniel den Hoed Kliniek) and Erasmus University Medical Center, ‘s-Gravendijkwal 230, Rotterdam, The Netherlands; 14 Department of Clinical Oncology, Leiden University Medical Center, Albinusdreef 2, Leiden, The Netherlands

**Keywords:** Cancer, Oncology, Prostatic neoplasms, Questionnaires, Testicular neoplasms

## Abstract

**Background:**

Understanding of the physical, functional and psychosocial health problems and needs of cancer survivors requires cross-national and cross-cultural standardization of health-related quality of life (HRQoL) questionnaires that capture the full range of issues relevant to cancer survivors. To our knowledge, only one study has investigated in a comprehensive way whether a questionnaire used to evaluate HRQoL in cancer patients under active treatment is also reliable and valid when used among (long-term) cancer survivors. In this study we evaluated, in an international context, the psychometrics of HRQoL questionnaires for use among long-term, disease-free, survivors of testicular and prostate cancer.

**Methods:**

In this cross-sectional study, we recruited long-term survivors of testicular and prostate cancer from Northern and Southern Europe and from the United Kingdom who had participated in two phase III EORTC clinical trials. Participants completed the SF-36 Health Survey, the EORTC QLQ-C30 questionnaire, the QLQ-PR25 (for prostate cancer) or the QLQ-TC26 (for testicular cancer) questionnaires, and the Impact of Cancer questionnaire. Testicular cancer survivors also completed subscales from the Nordic Questionnaire for Monitoring the Age Diverse Workforce.

**Results:**

Two hundred forty-two men (66% response rate) were recruited into the study. The average time since treatment was more than 10 years. Overall, there were few missing questionnaire data, although scales related to sexuality, satisfaction with care and relationship concerns of men without partners were missing in more than 10% of cases. Debriefing showed that in general the questionnaires were accepted well. Many of the survivors scored at the upper extremes of the questionnaires, resulting in floor and ceiling effects in 64% of the scales. All of the questionnaires investigated met the threshold of 0.70 for group level reliability, with the exception of the QLQ-TC26 (mean reliability .64) and the QLQ-PR25 (mean reliability .69). The questionnaires were able to discriminate clearly between patients with and without comorbid conditions.

**Conclusions:**

The currently available HRQoL questionnaires exhibit acceptable psychometric properties and were well received by patients, but additional efforts are needed to ensure that the full range of survivor-specific issues is assessed.

## Background

More than half of European patients diagnosed with cancer enjoy survival of five years or longer after primary diagnosis [1]. However, a long period of survival is not synonymous with a life free of physical and psychosocial health problems related to the cancer and/or its treatment. Studies investigating health-related quality of life (HRQoL) in long-term cancer survivors have shown that cancer-related health concerns can persist for years after initial treatment [2–4].

Increasing attention is being paid to the HRQoL of long-term cancer survivors (i.e., 10 years or longer after diagnosis) [2, 5–7]. Understanding of the physical, functional and psychosocial health problems and needs of cancer survivors requires cross-national and cross-cultural standardization of HRQoL questionnaires that capture the full range of issues relevant to cancer survivors [8]. To our knowledge, only one previous study has investigated in a comprehensive way whether questionnaires used to evaluate HRQoL in cancer patients under active treatment are also reliable and valid when used among (long-term) cancer survivors. This study addressed the psychometrics of the EORTC Prostate cancer module (QLQ-PR25) in a large population-based sample from Ireland [9].

Although our ultimate goal is to identify and/or develop HRQoL questionnaires relevant to a wide range of cancer survivor populations, for this study we focused on survivors of two important genitourinary cancers – testicular cancer (TCa) and prostate cancer (PCa) - treated in the context of two EORTC phase III clinical trials [10, 11]. TCa is a disease that affects young adults. At the turn of the current century, the cure rate for TCa was slightly greater than 90% [12], and in the ensuing years it has increased to 98% [13]. Prostate cancer (PCa) is the most prevalent cancer among men in Western, industrialized countries [14]. For men diagnosed with local or loco-regional PCa, the relative 10 year survival rate is 91% [15].

From previous studies we know that, although TCa survivors report a level of general HRQoL comparable to the general population, they are confronted with a number of specific health issues. A minority of TCa survivors experience long-term side-effects of their treatment, including fertility problems [16–20], peripheral neuropathy, ototoxicity, Raynaud phenomena, gastrointestinal symptoms, decreased pulmonary function, cardiovascular disease and secondary tumors [17, 19, 21–23]. Also, there is evidence of heightened levels of fatigue, anxiety, and cancer-related distress [24–27], practical problems related to obtaining insurance or bank loans [6, 12], and less satisfactory social contacts with friends and acquaintances [18]. It should be noted, that TCa survivors also report positive consequences of having had cancer, including emotional growth, greater appreciation of life, and stronger relationships with family and friends [28].

As is the case for TCa survivors, PCa survivors report equal or better overall HRQoL as compared to healthy controls, and generally do well in terms of psychological well-being [29]. However, almost 40% of PCa survivors expresses heightened fear of disease recurrence and psychological distress [5]. Further, advanced age in (prostate) cancer survivors is associated with worse HRQoL outcomes [30–32], and comorbidity serves as an additional risk [33–35]. Moreover, treatment of PCa can have a profound impact on urinary, sexual and bowel function [36, 37]. These specific, long-term HRQoL problems are, in part, treatment dependent [31, 38–41]. Other long-term sequelae of PCa are hypertension, cerebrovascular episodes, osteoporosis, and neuropathy [3, 42].

The objectives of this study were twofold: 1) to determine the feasibility of conducting HRQoL research among long-term cancer survivors treated in EORTC phase III clinical trials; and 2) to evaluate the psychometrics of questionnaires for assessing the HRQoL of long-term cancer survivors (>10 years disease free). In a previous paper [43] we reported on the feasibility of and challenges associated with conducting HRQoL investigations among long-term cancer survivors. In the current paper, we focus on the second objective.

## Methods

### Participants

Participants were long-term survivors of two European Organisation for Research and Treatment of Cancer (EORTC) Genito-Urinary Cancers Group phase III clinical trials, with no evidence of active disease. The PCa survivors were recruited from *Trial 22911* [11] (a collaboration with the EORTC Radiation Oncology Group, inclusion 1992–2001), which investigated post-surgical (adjuvant) irradiation of the prostate surgical bed versus ‘wait and see’. We recruited survivors from the Netherlands, Belgium, France, and Italy. Since patients had entered this trial with a median age of 65 years, at the time of data collection at least 50% of patients were very elderly or had died. Therefore, we recruited four additional patients aged 65–75 years, who were not part of this trial but received the same treatment from the clinics that were included in this clinical trial.

The TCa survivors were recruited from *Trial 30941/MRC TE20* [10], which investigated different regimen of bleomycin, etoposide, and cisplatin (BEP) (inclusion 1995–1998). We recruited survivors from the Netherlands, Italy, Norway, and the United Kingdom. Since this trial included only six Southern European survivors, 37 additional survivors, treated according to the same regimen (three cycles of BEP over 5 days), were recruited from Italy outside of the trial.

We believe that it was important to supplement our sample to compensate for the underrepresentation of certain subgroups in the available clinical trial samples (i.e., those aged 65–75 years in the case of PCa, and those from Southern Europe in the case of TCa). There is no reason to believe that the inclusion of these additional patients in the study sample would influence the psychometric properties of the questionnaires under investigation. They were all long-term survivors as well.

### Study design

Eligible survivors received an invitation letter signed by their treating physician, an informed consent form and the questionnaire battery. A reminder was sent after three weeks (see [43] for detailed information on patient recruitment and procedures). The study was approved by the institutional review boards of the participating hospitals, and informed consent was obtained from all individual participants included in the study. Data were collected and processed at The Netherlands Cancer Institute.

#### HRQoL questionnaires

We assessed HRQoL at 3 levels: (1) generic (the SF-36 Health Survey) [44]; (2) cancer-specific (the EORTC core questionnaire (QLQ-C30) [45] and the EORTC prostate or testicular cancer modules (QLQ-PR25 [46]; QLQ-TC26) [47]); and (3) cancer survivor-specific (the Impact of Cancer questionnaire, version 2 (IOCv2) [8, 48]). Table 1 displays the subscales of these questionnaires. Additional questions were posed regarding marital status, social economic status (SES), race, comorbidity (the Charlson Index and the International Prognostic Index (IPI)), work-related problems and problems related to obtaining health and life insurance, and a home mortgage loan [49]. For the TCa survivors only, we also used four subscales of the Nordic Questionnaire for Monitoring the Age Diverse Workforce (QPSNordic) that assess work motivation, job and life satisfaction, health and well-being, and self-efficacy [50]. Respondents were also asked to complete a 10-item debriefing questionnaire to identify any questions that were perceived as difficult to answer, confusing or upsetting, and to report important survivorship issues that were not (sufficiently) addressed by the questionnaires. All questionnaires had already been or were, for purposes of this study, translated using standard EORTC procedures [51].

**Table 1:** Questionnaire descriptive statistics and internal consistency reliability estimates for the prostate and testicular cancer survivor subsamples

	PCa Survivors					TCa Survivors				
Questionnaire scales	*N*	M	SD	% floor/ ceiling	Cronbach’sα	*N*	M	SD	% floor/ ceiling	Cronbach’s α
SF-36										
Physical Functioning	112	73	27		0.93	124	92	15	52c	0.91
Role-Physical	113	61	44	50c	0.91	125	89	26	77c	0.86
Bodily Pain	116	68	26	26c	0.91	126	85	21	56c	0.93
General Health	116	64	20		0.77	125	74	21		0.80
Vitality	114	64	20		0.82	123	66	19		0.82
Social Functioning	116	79	23	38c	0.86	126	84	22	54c	0.81
Role-Emotional	112	74	40	67c	0.88	126	90	24	80c	0.68
Mental Health	113	75	17		0.80	123	79	15		0.83
IOCv2										
Altruism/empathy	115	3.3	0.7		0.77	125	3.5	0.8		0.77
Health awareness scale	114	3.1	0.9		0.74	125	3.4	0.8		0.71
Meaning of cancer scale^a^	112	2.6	0.8		0.85	110	2.8	0.8		0.82
Positive self-evaluation	115	3.3	0.7		0.64	126	3.5	0.8		0.69
Positive Impact Score	115	3.1	0.5		0.82	126	3.3	0.6		0.87
Appearance concerns	114	1.8	0.7	25f	0.73	124	1.9	0.9	33f	0.83
Body change concerns	115	2.6	1.0		0.81	124	2.2	0.9		0.77
Life interferences	115	2.1	0.7		0.75	126	1.8	0.8	21f	0.85
Worry	115	2.4	0.9		0.90	125	2.4	0.9		0.90
Negative Impact Score	115	2.2	0.7		0.93	126	2.1	0.7		0.94
Employment concerns^b^	132	2.7	1.1		0.63	111	2.1	0.9	23f	0.74
Relationship/not partnered concerns	25	2.3	0.9		0.82	24	2.2	1.1	25f	0.85
Relationship/partnered concerns	92	1.8	0.6	22f	0.52	102	1.5	0.6	39f	0.62
QLQ-C30										
Global health status/QoL	112	69	20		0.96	124	79	19	22c	0.92
Physical Function	113	84	18	30c	0.83	126	95	12	68c	0.82
Role Function	114	85	24	61c	0.90	126	94	17	84c	0.93
Emotional Function	113	85	18	40c	0.84	124	86	17	39c	0.84
Cognitive Function	114	83	21	44c	0.73	124	91	16	66c	0.61
Social Function	114	88	20	61c	0.78	124	94	16	82c	0.86
Fatigue	114	22	21	28f	0.84	126	21	21	33f	0.85
Nausea/vomiting^c^	114	1	5	91f	−0.04	126	2	6	87f	–
Pain	114	18	25	53f	0.89	126	12	20	63f	0.85
Dyspnoea^d^	114	15	22	63f	–	125	9	18	75f	–
Insomnia^d^	113	18	29	65f	–	126	13	22	68f	–
Appetite loss^d^	113	5	17	91f	–	126	4	14	89f	–
Constipation^d^	114	11	21	75f	–	120	5	14	87f	–
Diarrhoea^d^	113	7	18	83f	–	124	6	17	85f	–
Financial problems^d^	114	5	18	90f	–	123	8	21	86f	–
QLQ-PR25										
Sexual activity	112	26	25	32 f	0.67	–	–	–	–	–
Sexual functioning	43	51	20		0.41	–	–	–	–	–
Urinary problems	115	22	20		0.89	–	–	–	–	–
Incontinence aid problems^d^	49	29	34	49 f	–	–	–	–	–	–
Bowel problems	108	8	14	52 f	0.79	–	–	–	–	–
Treatment related symptoms	112	13	12		0.45	–	–	–	–	–
QLQ-TC26										
Satisfaction with care	–	–	–	–	–	111	80	28	52c	0.85
Future perspective	–	–	–	–	–	125	77	24	36c	0.56
Communication	–	–	–	–	–	125	76	25	34c	0.53
Sexual activity	–	–	–	–	–	121	60	27		0.77
Sexual enjoyment	–	–	–	–	–	116	76	25	38c	0.77
Satisfaction with testicular implant^d^	–	–	–	–	–	35	55	37	31c	–
Treatment side effects	–	–	–	–	–	125	11	12	28f	0.63
Job problems	–	–	–	–	–	125	15	26	66f	0.82
Sexual problems	–	–	–	–	–	115	13	22	65f	0.59
Family problems^d^	–	–	–	–	–	125	15	28	70f	–
Infertility^d^	–	–	–	–	–	123	24	34	58f	–
Body Image^d^	–	–	–	–	–	125	18	28	62f	–
QPSNordic										
Work Motivation	–	–	–	–	–	113	3.8	0.7		0.42
Job and life satisfaction	–	–	–	–	–	113	3.9	0.8		0.71
Work ability	–	–	–	–	–	113	3.8	0.8		0.82
Self-efficacy	–	–	–	–	–	113	4.4	0.8	40c	0.83

Scale range for the SF-36, QLQ-C30, QLQ-PR25 and QLQ-TC26 is from 0 to 100. The IOCv2 and QPS Nordic scale range from 1 to 5. For the function scales and global health scale of the EORTC-QLQC30 and its modules a higher score represents a better level of functioning. For the symptom oriented scales and items higher scores indicate a higher level of symptoms; Percentage floor or ceiling effects are only reported when they exceed 20%.; *TCa* testicular cancer survivors, *PCa* prostate cancer survivors, *N* number of participants, *M* mean, *SD* standard deviation, *c* ceiling, *f* floor

^a^Due to a mistake in the Norwegian version of the IOCv2 14 respondents skipped more than half of the items of this scale

^b^N is higher than the number of respondents who were actually employed, since the item was also completed by some participants who were retired

^c^Reliability of the NV scale could not be calculated do to the very low prevalence of nausea and the absence of vomiting in the TCa sample

^d^Single item scales

### Statistical analysis

We performed missing data analyses at both the item and scale level. A scale score was defined as missing if more than half of the items in that scale were missing. In all other cases, we generated a person-specific scale score based on the mean of the non-missing items [52, 53]. For scales for which scores could not be calculated for 5% or more of respondents, we conducted logistic regression analysis to determine if missingness was associated significantly with country/language, age, education or marital status.

Descriptive statistics were generated for all measures, including means, standard deviations and floor and ceiling effects (20% or more of the scores at the extremes of the scale [54]). We used Cronbach’s coefficient α [55] to estimate the reliability (internal consistency) of the questionnaire scales.

We employed analysis of variance to investigate known groups validity, i.e., the extent to which the questionnaire scores are able to discriminate between relevant predefined subgroups. Grouping variables included age, education, marital status, disease site (testicular versus prostate) and comorbidity. A sample size of 120 is sufficient to detect effect size differences of .25 in a 2-group design and .30 in a 3-group design with a power of .80 and an α .05. An effect size of .25 is the equivalent of a one-quarter standard deviation difference between groups in mean scores. An effect size of 0.2 is interpreted as a small difference, 0.5 as a moderate difference, and 0.8 as a large difference [56]. The *a priori* hypotheses were that those who were younger, with a higher education, with a partner, and with fewer comorbid conditions (as assessed by self-report) would generally report better HRQoL. We also hypothesized that TCa survivors would report better HRQoL than PCa survivors, due to their younger age and to the relatively transient nature of some of the treatment effects.

## Results

### Sample accrual and response rate

Data collection took place between February 2010 and June 2012. Of the 366 survivors invited to participate in the study, 242 (66%) agreed to do so. The response rate in the PCa survivor group was somewhat higher than in the TCa group (69% vs 64%). See van Leeuwen et al. [43] for more details on patients recruitment. Table 2 reports the patient background characteristics.

**Table 2:** Sample characteristics of the prostate and testicular cancer survivor samples

	PCa survivors (*N* = 116)		TCa survivors (*N* = 126)	
Age				
Mean ± SD (years)	75.0	(5.8)	43.1	(8.8)
Civil status (%)				
married/living with partner	92	(79%)	85	(67%)
widower	11	(10%)	–	
divorced/separated	8	(7%)	7	(6%)
in relationship living apart	–		15	(12%)
never been married/living together	4	(3%)^a^	16	(13%)
Education (%)				
university	23	(20%)	42	(33%)
vocational education	46	(40%)	50	(40%)
high school	24	(21%)	26	(21%)
primary school	21	(18%)	6	(5%)
Work status (%)				
fulltime (≥ 32 h per week)	2	(2%)	92	(73%)
part-time (< 32 h per week)	1	(1%)	13	(10%)
irregular hours	1	(1%)	–	
retired	111	(96%)	6	(5%)
unemployed	–		2	(2%)
incapable of work	1	(1%)	9	(7%)
household	–		2	(2%)
Country (%)				
The Netherlands	44	(38%)	37	(29%)
Belgium	37	(32%)	–	
United Kingdom	–		29	(23%)
Norway	–		23	(18%)
France	25	(22%)	–	
Italy	10	(9%)	37	(29%)
Time since treatment allocation				
Mean (SD) (years)	13.0	(2.1)	–	
Time since treatment evaluation				
Mean (SD) (years)	–		11.9	(3.8)
Treatment allocation (%)				
prostatomectomy and wait and see	53	(46%)	–	
prostatomectomy and radiation	61	(53%)	–	
four cycles/ five days	–		27	(21%)
four cycles/ three days	–		23	(18%)
three cycles/ five days	–		53	(42%)^b^
three cycles/ three days	–		23	(18)
Co-morbid disorders (%)				
0	22	(19%)	58	(46%)
1	22	(19%)	36	(29%)
2	29	(25%)	20	(16%)
3 or more	35	(30%)	8	(6%)

^a^Totals which do not add up to 100% are caused by missing data. ^b^Most patients recruited outside the trial were treated with this regimen. *N* number of participants, *SD* Standard deviation

### Completeness of the data

With a few exceptions, the percentage of missing item values was low, ranging from 0 to 7%. Although TCa survivors had to complete more items, items were missing less frequently in the TCa sample than in the PCa sample. The most important exceptions were the missing responses on the items of the Sexual Functioning scale of the QLQ-PR25. Among the PCa survivors who had indicated that they were sexually active during the past week, missing responses varied between 37 and 43%. Other QLQ-PR25 items with relatively high percentages of missing responses were one item from the Urinary Symptom scale (posed following an item which was only applicable to patients wearing incontinence material; 14%), the Sexual Activity scale (10%), and items from the Treatment-Related Symptom scale that were related to hormonal treatment (6 to 8%). For the IOCv2, the highest percentage of missing responses was for the Relationship Concerns (both partnered and not partnered) scales (7 to 23%).

There were four scales for which we could not calculate the scale scores for more than 5% of the PCa sample: the QLQ-PR25 Sexual Functioning and Bowel Symptom scales (37 and 7%, respectively); and the IOCv2 Relationship Concern scales (both partnered and not partnered) (20 and 8%, respectively). Missing responses on the QLQ-PR25 Sexual Functioning scale were associated significantly with (older) age. Belgian participants were significantly more likely to skip items of the Bowel Symptom scale. No other significant associations were observed in the PCa sample between item missingness and sociodemographic variables.

In the TCa sample, missing responses were primarily observed for those items that were associated with contingency questions (e.g., the IOCv2 items applicable only for sub-groups of respondents, such as those with a partner). With the exception of the item “I wonder how to tell a potential spouse, partner, boyfriend or girlfriend that I have had cancer” (skipped by 18% of the respondents), missing item responses for those scales varied between 4 and 8%. Items of the QLQ-TC26 that were related to satisfaction with care (14%) and to sexuality (6 to 10%) were also skipped more frequently.

There were four scales for which scores could not be calculated for more than 5% of the TCa sample: the IOCv2 Relationship Concerns scale (not partnered; 13%); and Satisfaction with Care scale (12%), the Sexual Enjoyment (5%), and Sexual Problems (6%) of the QLQ-TC26. The Italian patients were most likely to complete the Satisfaction with Care and the Sexual Problems scale. Items from the Sexual Enjoyment and Sexual Problems scales were most frequently left unanswered by respondents without a partner. Dutch respondents also skipped more items of the Sexual problems scale.

### Debriefing questionnaire

Sixty-five survivors (of whom 27 were from the TCa sample) reported an issue with one or more of the questions or left a general comment for the doctors or researchers on the debriefing questionnaire. Twelve survivors stated that they had difficulties distinguishing cancer-related problems from those due to aging and other conditions. Seven survivors reported they found the questions regarding sexual problems too personal, too confusing and/or too difficult to answer. Two survivors found the questions regarding incontinence too confusing or too difficult. Three survivors found that some questions were more related to the treatment and diagnosis phase. Further, 18 survivors reported missing issues, of which dealing with sexual problems (6) and long-term side effects of treatment (2) were the most often mentioned. Finally, four survivors over 80 years of age considered some questions redundant for survivors of their age (e.g. questions regarding work, feeling old, and future perspective).

### Scale level descriptive statistics and reliability

Scale level descriptive statistics and reliability estimates are presented in Table 1.

#### The SF-36

Ceiling effects were observed for four of the eight SF-36 scales in the PCa sample, and for five scales in the TCa sample. The role functioning scales exhibited the most serious ceiling effects. Cronbach’s coefficient *α* varied between .68 and .93 (see Table 1).

#### The EORTC QLQ-C30

All scales of the EORTC QLQ-C30 exhibited ceiling or floor effects in both the PCa and the TCa, samples, with the exception of the Global quality of life scale in the PCa sample. Cronbach’s *α* coefficients varied between .70 and .96, with the exception of the Emesis scale, which was very low in the PCa sample (0.04) and could not be calculated in the TCa sample due to the extremely low prevalence rates for these symptoms in these survivor samples (see Table 1).

#### The IOCv2

The Relation Concerns (partnered) and the Appearance Concerns scales exhibited floor effects in both samples. In the TCa sample, the Life Interferences, Employment Concerns and Relationship Concerns (not partnered) scales exhibited significant floor effects. Cronbach’s *α* coefficients ranged from .52 to .94. Three of the 11 IOCv2 scales had a Cronbach’s *α* lower than .70 in the PCa sample (Positive self-evaluation (.64), Employment concerns (.63) and Relationship partnered concerns (.52)), and two scales had a Cronbach’s *α* coefficient lower than .70 in the TCa sample (Positive self-evaluation (.69) and Relationship partnered concerns (.62); see Table 1).

#### The QLQ-PR25

Three of the six scales of the QLQ-PR25 exhibited a floor effect. Cronbach’s *α coefficient* varied between .41 and .89, with three scales below .70 (Sexual activity (.67), Treatment related symptoms (.45) and Sexual functioning (.41)) (see Table 1).

#### The QLQ-TC26

Except for Sexual Activity, all scales of the QLQ-TC26 showed substantial floor and ceiling effects. Cronbach’s *α* coefficient varied between .53 and .89, with four scales scoring below .70. The Cronbach’s *α* for Future perspective was .56, for Communication .53, for Treatment side effects .63, and for Sexual Problems .59 (see Table 1).

#### The QPSNordic

Only the Self-Efficacy scale of the QPSNordic showed a substantial ceiling effect. Cronbach’s *α coefficient* varied between .42 and .83, with only Work motivation being below .70 (see Table 1).

### Tests of known-groups validity

#### Age

In the PCa sample, (older) age was most strongly associated with physical health problems, although there was also a strong association with cognitive functioning and sexual activity level. Effects sizes ranged from 0.32 to 1.05. The association between age and HRQoL was much less pronounced in the TCa than in the PCa sample. However, the younger men in the TCa sample reported significantly more mental health problems (effect size (ES) .37–.58), were more sexually active (ES .17–.66) and worried significantly more about their fertility (ES.70–1.07) than older men (see Tables 3 and 4).

**Table 3:** Known group validity testing: Effect sizes for the differences within the prostate cancer sample between subgroups formed on the basis of comorbidity status, age, education and marital status

	PCa survivors									
	Co-morbidities		Age			Education			Marital status	
	0–1 comorbidities^a^	2 or more co-morbidities	Younger than 70^a^	70–80	80 and older	University^a^	College and high school	Primary school only	Single^a^	Partner
SF36: Physical Functioning		**−0.60**		**−0.72**	**−1.05**		−0.39	−0.67		0.22
SF36: Role-Physical		**−0.66**		**−0.66**	**−0.84**		−0.43	−0.44		−0.07
SF36: Bodily Pain		**−0.86**		−0.32	−0.55		−0.50	−0.53		−0.03
SF36: General Health		**−0.48**		**−0.42**	**−0.73**		−0.42	−0.70		0.30
SF36: Vitality		**−0.57**		−0.40	−0.58		**−0.54**	**−0.73**		0.03
SF36: Social Functioning		**−0.47**		−0.30	−0.41		−0.38	−0.56		0.27
SF36: Role-Emotional		**−0.44**		−0.46	−0.61		−0.56	−0.58		0.25
SF36: Mental Health		**−0.49**		−0.29	−0.65		**−0.47**	**−1.02**		0.26
QLQ-C30: Global health status/QoL		**−0.61**		**−0.51**	**−0.85**		**−0.51**	**−0.79**		−0.01
QLQ-C30: Physical Function		**−0.58**		**−0.68**	**−0.95**		−0.34	−0.63		0.12
QLQ-C30: Role Function		**−0.52**		−0.50	−0.55		−0.31	−0.59		−0.05
QLQ-C30: Emotional Function		**−0.51**		−0.27	−0.35		**−0.35**	**−1.01**		0.27
QLQ-C30: Cognitive Function		**−0.49**		**−0.41**	**−0.87**		−0.30	−0.48		0.03
QLQ-C30: Social Function		**−0.58**		−0.15	−0.29		−0.19	−0.53		0.37
QLQ-C30: Fatigue		**−0.57**		−0.23	−0.25		**−0.53**	**−0.86**		0.02
QLQ-C30: Nausea / vomiting		−0.14		0.07	−0.27		−0.10	−0.55		0.21
QLQ-C30: Pain		**−0.58**		−0.34	−0.40		−0.32	−0.27		−0.08
QLQ-C30: Dyspnoea		**−0.54**		−0.30	−0.60		−0.16	−0.65		0.34
QLQ-C30: Insomnia		−0.34		−0.39	−0.43		−0.32	−0.50		0.20
QLQ-C30: Appetite loss		−0.05		−0.23	0.29		−0.14	−0.63		0.09
QLQ-C30: Constipation		**−0.50**		**−0.03**	**−0.57**		**−0.05**	**−0.66**		0.04
QLQ-C30: Diarrhoea		−0.23		0.32	0.75		−0.07	0.16		0.32
QLQ-C30: Financial problems		0.12		0.63	0.35		−0.16	−0.21		0.36
IOC: Appearance concerns		**−0.46**		−0.38	−0.18		**−0.25**	**−0.94**		0.35
IOC: Body change concerns		**−0.55**		−0.41	−0.18		−0.42	−0.55		0.09
IOC: Life interferences		**−0.59**		−0.16	−0.28		**−0.54**	**−0.84**		0.16
IOC: Worry		**−0.46**		0.03	0.05		−0.05	−0.41		0.19
IOC: Negative Impact Scale		**−0.62**		−0.19	−0.15		−0.35	−0.73		0.21
PR25: Sexual activity		−0.12		**−0.32**	**−0.75**		0.05	−0.18		0.38
PR25: Sexual functioning		−0.51		−0.63	−0.35		0.25	0.22		0.23
PR25: Urinary problems		**−0.50**		−0.37	−0.60		−0.47	−0.67		0.27
PR25: Incontinence aid problems		−0.36		−0.74	−0.27		−0.43	−0.71		−0.30
PR25: Bowel problems		−0.39		−0.30	−0.51		−0.02	−0.61		0.16
PR25: Treatment related symptoms		−0.34		−0.27	−0.51		−0.54	−0.44		−0.01

Effect sizes are displayed in bold in those cases where the known groups differed significantly (*p* ≤ .05). A positive effect size reflects better HRQoL than the reference group, and a negative effect size reflects worse HRQoL than the reference group. ^a^reference group. PCa = prostate cancer

**Table 4:** Known group validity testing: effect sizes (ES) of the differences within the testicular cancer sample between the known groups based on comorbidity status, age, education and marital status

	TCa survivors									
	Co-morbidities		Age			Education			Marital status	
	0 comorbidities^a^	1 or more co-morbidities	Younger than 40^a^	40–50	50 and older	University^a^	Higher education	High school or less	Single^a^	Partner
SF36: Physical Functioning		**−0.66**		−0.33	−0.41		**−0.11**	**−0.58**		**0.61**
SF36: Role-Physical		**−0.62**		−0.09	−0.12		−0.20	−0.24		**0.93**
SF36: Bodily Pain		**−0.84**		−0.34	−0.28		0.00	−0.14		**0.64**
SF36: General Health		**−0.55**		−0.37	−0.21		0.03	−0.16		**0.74**
SF36: Vitality		**−0.61**		−0.07	−0.13		0.13	−0.10		**0.52**
SF36: Social Functioning		**−0.49**		−0.10	0.17		−0.18	−0.10		**0.93**
SF36: Role-Emotional		−0.27		0.06	0.07		−0.21	−0.24		**0.64**
SF36: Mental Health		−0.20		**0.37**	**0.58**		−0.18	−0.11		**0.65**
QLQ-C30: Global health status/QoL		**−0.36**		−0.09	0.20		0.01	−0.29		**0.67**
QLQ-C30: Physical Function		**−0.69**		−0.29	−0.27		0.07	−0.42		**0.75**
QLQ-C30: Role Function		**−0.55**		−0.19	0.08		0.04	−0.43		**0.90**
QLQ-C30: Emotional Function		**−0.48**		0.01	0.17		0.04	−0.26		**0.86**
QLQ-C30: Cognitive Function		**−0.48**		−0.07	−0.30		−0.06	0.08		**0.74**
QLQ-C30: Social Function		**−0.47**		−0.07	0.12		0.14	0.01		**0.66**
QLQ-C30: Fatigue		**−0.81**		−0.07	−0.38		0.12	−0.31		**0.76**
QLQ-C30: Nausea / vomiting		**−0.40**		0.12	0.17		−0.12	−0.25		**0.55**
QLQ-C30: Pain		**−0.80**		−0.20	−0.19		0.13	−0.13		**0.63**
QLQ-C30: Dyspnoea		**−0.45**		−0.21	−0.40		**0.27**	**−0.37**		0.19
QLQ-C30: Insomnia		**−0.43**		0.15	0.14		−0.03	−0.04		**0.73**
QLQ-C30: Appetite loss		−0.17		0.06	0.10		0.04	−0.33		**0.60**
QLQ-C30: Constipation		−0.24		−0.10	−0.03		0.31	0.06		**0.48**
QLQ-C30: Diarrhoea		−0.03		−0.13	0.04		**0.59**	**0.56**		0.15
QLQ-C30: Financial problems		**−0.37**		0.06	−0.40		0.09	−0.12		0.41
IOC: Appearance concerns		−0.13		0.19	0.35		0.07	−0.07		0.31
IOC: Body change concerns		**−0.41**		−0.15	−0.32		−0.06	−0.30		0.34
IOC: Life interferences		−0.33		−0.01	−0.02		−0.07	−0.14		**0.65**
IOC: Worry		**−0.42**		−0.06	0.12		0.02	−0.19		0.23
IOC: Negative Impact Scale		**−0.42**		−0.04	0.04		−0.02	−0.25		**0.50**
TC26: Satisfaction with care		−0.23		−0.08	−0.17		0.02	0.03		−0.06
TC26: Future perspective		−0.32		0.01	0.07		−0.04	−0.36		**0.52**
TC26: Communication		0.05		0.16	−0.16		−0.22	−0.04		0.04
TC26: Sexual activity		−0.30		**−0.17**	**−0.66**		−0.19	−0.13		**0.56**
TC26: Sexual enjoyment		−0.30		0.00	−0.54		−0.15	−0.27		**0.57**
TC26: Satisfaction with testicular implant		−0.36		0.76	0.50		−0.09	−0.78		0.26
TC26: Treatment side effects		**−0.57**		−0.10	−0.08		−0.04	−0.14		**0.52**
TC26: Job problems		−0.34		−0.01	0.15		−0.02	−0.31		**0.48**
TC26: Family problems		−0.17		−0.05	0.07		0.18	−0.18		0.09
TC26: Infertility		0.06		**0.70**	**1.07**		0.14	0.04		0.23
TC26: Body Image		−0.26		0.31	0.14		0.36	0.01		**0.54**
TC26: Sexual problems		**−0.38**		0.17	−0.23		−0.19	−0.06		0.36
QPS: Work Motivation		−0.28		−0.05	0.49		−0.03	0.03		**0.49**
QPS: Job and life satisfaction		−0.21		−0.01	0.21		−0.03	0.05		**0.51**
QPS: Work ability		−0.37		−0.23	−0.50		−0.26	−0.37		**0.59**
QPS: Self-efficacy		−0.13		−0.41	−0.37		−0.28	−0.27		−0.27

Effect sizes are displayed in bold in those cases where the known groups differed significantly (*p* ≤ .05). *TCa* testicular cancer

A positive effect size reflects better HRQoL than the reference group, and a negative effect size reflects worse HRQoL than the reference group

^a^reference group

#### Education

In the PCa sample, higher education was significantly associated with better physical and mental health, less appearance concerns and life interferences (effect sizes ranging from .25 to greater than 1). There was only a weak association between education level and HRQoL observed in the TCa sample (see Tables 3 and 4).

#### Marital status

Marital status was not significantly associated with HRQoL in the PCa sample. In contrast, in the TCa sample, single men reported consistently and significantly worse HRQoL than partnered men across almost all scales of all questionnaires, with effect sizes ranging from .48 to .93 (see Tables 3 and 4).

#### Comorbidity

In both the PCa and the TCa samples, men with two or more comorbid conditions (or one or more in the TCa sample) reported significantly and consistently poorer HRQoL, as measured by all questionnaires, than those without comorbid conditions. Effect sizes (ES) ranged from .36 to .86 (see Tables 3 and 4).

#### PCa versus TCa sample

As hypothesized, the TCa sample reported significantly better physical health, social and cognitive functioning, and fewer symptoms than the PCa sample, with effect sizes ranging from .29 to .82. No significant group differences were observed for fatigue, social functioning, mental health, or for most of the symptom scales of the QLQ-C30 (see Table 5).

**Table 5:** Known group validity testing: effect sizes of the differences between the prostate and the testicular cancer samples

PCa versus TCa survivors	
SF36: Physical Functioning	**0.82**
SF36: Role-Physical	**0.73**
SF36: Bodily Pain	**0.69**
SF36: General Health	**0.51**
SF36: Vitality	0.10
SF36: Social Functioning	0.23
SF36: Role-Emotional	**0.48**
SF36: Mental Health	0.25
QLQ-C30: Global health status/QoL	**0.49**
QLQ-C30: Physical Function	**0.67**
QLQ-C30: Role Function	**0.44**
QLQ-C30: Emotional Function	0.01
QLQ-C30: Cognitive Function	**0.40**
QLQ-C30: Social Function	**0.33**
QLQ-C30: Fatigue	0.06
QLQ-C30: Nausea / vomiting	−0.16
QLQ-C30: Pain	**0.30**
QLQ-C30: Dyspnoea	**0.29**
QLQ-C30: Insomnia	0.18
QLQ-C30: Appetite loss	0.01
QLQ-C30: Constipation	**0.31**
QLQ-C30: Diarrhoea	0.05
QLQ-C30: Financial problems	−0.02
IOC: Appearance concerns	−0.03
IOC: Body change concerns	**0.38**
IOC: Life interferences	**0.45**
IOC: Worry	−0.01
IOC: Negative Impact Scale	0.21

Effect sizes are displayed in bold in those cases where the known groups differed significantly (*p* ≤ .05). *PCa* prostate cancer, *TCa* testicular cancer. A positive effect size reflects better HRQoL in TCa survivors

## Discussion

In this study we have assessed the psychometric performance of a number of widely used HRQoL questionnaires for specific use in cancer survivorship research. The response rate was quite reasonable (66%), given that these were long-term survivors (on average, more than 10 years post-diagnosis and treatment). Our response rate is comparable to that of many mail surveys in health research [57, 58]. The questionnaires were well received by these survivors.

### Psychometric performance of the questionnaires

Missing items were particularly an issue for the scales assessing employment and relationship concerns in the IOCv2, and the scales of the cancer-specific modules assessing sexuality, bowel functioning, complaints related to hormonal treatment (for PCa) and treatment satisfaction. This problem with missing item responses was often related to the sensitive nature of the questions or to branching and skip patterns in the questionnaire (i.e., use of contingency questions). The high level of missingness in the scales assessing sexual problems and functioning are particularly noteworthy, and this could lead to an underestimation of the prevalence of these problems in these survivor populations. Missing responses to sensitive questions (e.g. sexuality) are common and quite difficult to resolve, although there are studies suggesting that response rates could be improved by using online computer administration [59]. Further, our findings suggest that branching questions should be used judiciously to minimize respondent confusion.

The questionnaires assessing symptoms and physical functioning all showed floor and ceiling effects, particularly in the younger TCa survivors. This can be expected in groups of survivors where the self-reported HRQoL is similar to that of the healthy, general population. Moreover, short-term, treatment-induced symptoms like nausea and vomiting or acute hair loss often are no longer relevant in these long-term survivors. These floor and ceiling effects are due, at least in part, to the fact that the SF-36 and the EORTC QLQ-C30 and its condition-specific modules were not developed for or intended to discriminate between individuals who are at the higher end of the health spectrum (e.g., those with moderate to excellent health). A number of studies have previously reported that the SF-36 and the EORTC QLQ-C30 exhibit significant ceiling and floor effects when used in general population surveys [60–63].

In general, the internal consistency reliability of the questionnaires was acceptable for group level comparisons. Problems were observed with several scales of the PR25 and of the TC26, two scales of the IOCv2, and one scale of the QPSNordic. In the case of the treatment-related symptom scales, this often reflected the low prevalence of certain symptoms among long-term survivors, as has also been reported by O’Leary in a population-based sample of prostate cancer survivors [9]. However, suboptimal reliability has also been reported for some scales even during the period of active treatment [46]. The problematic internal consistency of the positive self-evaluation scale of the IOCv2 was also observed in an Italian validation study [64] and in a French validation study the health awareness scale appeared problematic [65]. However, in a Dutch validation sample none of these problems were observed [66].

The HRQoL questionnaires were able to discriminate well between patients with and without comorbid conditions. Comorbid disorders might affect self-reported functioning more strongly than any residual effect from cancer or its treatment [61]. In the other known-group comparisons there was not always sufficient power to reach statistical significance. Nevertheless, the trends observed in the known groups were compatible with our hypotheses, except for the higher levels of mental health problems reported by the younger TCa survivors. This latter finding is similar to results reported in studies of younger breast cancer survivors [67–70]. The recent study of Drummond et al. shows that, with larger sample sizes, the EORTC QLQ-C30 and the QLQ-PR25 are able to discriminate between different initial treatment modalities in long-term prostate cancer survivors [71].

### Recommendations for assessing HRQOL in cancer survivors

Despite their limitations, we consider both the SF-36 and the EORTC QLQ-C30 to be sufficiently robust psychometrically to be employed in cancer survivorship studies. Although they may not be able to differentiate clearly between survivors at higher levels of functioning and lower levels of symptom burden, they can identify functional health issues and relevant symptom levels that may require attention by health care providers.

The IOCv2 is a questionnaire which addresses the unique concerns related to the cancer experience that are not captured by the more generic HRQoL questionnaires [48]. Most of the previous studies using the IOCv2 have been carried out in mixed samples and samples with women only e.g. [66, 72], which makes their results difficult to compare with ours. In particular, we know that women report more extreme impacts of cancer and its treatment (both positive and negative) on their lives than men [72, 73]. We can best compare our results with two previous studies of male hematological survivors and with the Norwegian validation study [66, 73, 74]. Compared to long-term male lymphoma survivors, survivors in our sample reported less extreme negative or positive impacts of having had cancer [74]. Similar to what has been reported previously by Oerlemans et al. [66], the men in our sample reported few appearance concerns, and, like Dahl et al. [73], we found that TCa survivors were less negatively impacted by cancer than PCa survivors. These differences in outcomes on the IOCv2 underline the relevance of this questionnaire for detecting differences between survivor groups in how cancer and its treatment have affected their lives.

The selected scales from the QPSNordic can be particularly useful in assessing cancer survivors’ work experience. Ours as well as previous studies (e.g. [72, 75, 76]) indicate that a significant minority of cancer survivors experience work-related changes after being diagnosed with and treated for cancer. Particularly survivors with mental and physical health problems are vulnerable to work-related problems. Work ability levels observed in our sample of TCa survivors were comparable to that of mid-term Norwegian breast, TCa, and PCa cancer survivors. Gudbergsson et al. reported that the work ability in these cancer survivors was significantly worse than that of an age and gender matched control group [77].

The Patient-Reported Outcomes Measurement Information System (PROMIS) initiative [78] and the EORTC Quality of Life Group (EORTC QLG) Computer-Adaptive Testing project [79, 80] are currently developing HRQoL instruments based on item response theory which, among other things, will make it possible to discriminate better between individuals who score at the extremes of the health continuum. Also, the EORTC QLG is currently developing an HRQoL assessment strategy specifically for disease-free cancer survivors, focusing on longer-term physical effects of cancer and its treatment, psychological aspects of survivorship, and important issues such as fear of recurrence and health awareness, and excluding acute treatment related symptoms.

### Generalizability of our findings

Even though our response rate is quite reasonable for this type of long-term follow-up study, we cannot rule out the possibility of some degree of selection bias in our sample. It could be that the non-respondents had fewer physical and psychosocial health issues (and thus were less motivated to participate in the study) than those who participated. It may also be that non-respondents did not want to be confronted with their earlier cancer experience. Unfortunately, we do not have any information about the actual reasons for not participating in the study, and thus the nature of any bias, if present, remains unclear.

As our study sample was restricted to male survivors only, we cannot generalize our findings to the larger population of cancer survivors. Additional studies are needed that include significant numbers of female cancer survivors. There is, for example, some evidence that women report higher levels of symptom burden and functional impairment than men. Problems observed in the current study with regard to floor and ceiling effects may be less pronounced in studies of female survivors or with mixed samples with regard to sex [62].

Additionally, our study sample was composed primarily of participants in randomized clinical trials. Patients treated in the context of clinical trials often are not entirely representative of the patient population encountered in clinical practice. Trial patients are often younger and have fewer comorbid conditions than non-trial patients [81, 82]. Also, patients treated in the context of a trial may receive more consistent and higher quality care than those treated outside of a trial context [83]. While this may affect the actual prevalence of symptoms and functional limitations reported by long-term survivors, it is less relevant for evaluating the psychometric properties of HRQoL questionnaires, which was the focus of our study. Also, given the relatively long period of time since diagnosis and active treatment, the effect of having been treated in the context of a clinical trial probably has less impact on the generalizability of findings than might be the case in a study with a shorter follow-up [81, 82]. In any case, further studies are needed that investigate the performance of these and other HRQoL questionnaires when used with the broader clinic populations.

## Conclusions

In general, the questionnaires evaluated in this study were found to be reliable and valid when used among male cancer survivors in various European settings. Several international initiatives are developing both computer-adaptive and survivorship-specific patient-reported outcome measures. These newer measures promise greater measurement precision and specificity. However, until they are sufficiently mature for general use, our results indicate that both the SF-36, the EORTC QLQ-C30 (and its modules), the IOCv2 and the QPSNordic are, within limits, useful tools for assessing the HRQoL of long term cancer survivors.

## Abbreviations

BEP: Bleomycin, etoposide, and cisplatin
EORTC: The European Organisation for Research and Treatment of Cancer
ES: Effect size
HRQoL: Health-related quality of life
IOCv2: The Impact of Cancer questionnaire, version 2
M: Mean
N: Number of participants
PCa: Prostate cancer
PROMIS: Patient-Reported Outcomes Measurement Information System
QLG: Quality of Life Group
QLQ-C30: The EORTC core questionnaire
QLQ-PR25: The EORTC prostate cancer module
QLQ-TC26: The EORTC testicular cancer module
QPSNordic: Nordic Questionnaire for Monitoring the Age Diverse Workforce
SD: Standard deviation
SES: Social economic status
SF-36: 36-Item Short Form Survey
TCa: Testicular cancer
